# Supplementary material for: Comparison of the Efficacy and Welfare of Different Training Methods in Stopping Chasing Behavior in Dogs
Source: Animals (Basel). 2024 Sep 11;14(18):2632. doi: 10.3390/ani14182632 (PMC11428818; doi:10.3390/ani14182632)
Supplement: Supplementary file 1 [file animals-14-02632-s001.zip › animals-3187886-supplementary.pdf]

Supplemental

Table S1. List of Questions and Possible Responses for Survey to Assess Interest in Study Participation.

**Question**	**Response**
Please provide your email	*Free response*
Zip code where you reside	*Free response*
Would you potentially be interested in traveling with your dog to participate in a study, located in Plant City, Florida	Yes<br>No
How many dogs do you own	1<br>2<br>3<br>4+
Dog Name	Puppy less than 6 months<br>6 months-1 year<br>1 year – 2 years<br>2 years- 3 years<br>3 years- 5 years<br>5 years – 7 years<br>7 years – 9 years<br>9+ years
Dog Breed	*Free response*
Does your dog chase things	Never<br>Sometimes<br>About half the time<br>Most of the time<br>Always
What types of items has your dog chased? (Select all that apply)	Moving vehicles<br>Bikes<br>Small animals (e.g.: cats, birds, rodents)<br>Deer<br>Toys (e.g.: balls, flirt piles<br>Other
Have you trained your dog with an electronic collar (e-collar)?	Never<br>Sometimes<br>About half the time<br>Most of the time<br>Always
Please select all that apply for why you have used an e-collar	Barking<br>Invisible Fence<br>Avoidance training (e.g.: rattlesnake avoidance"<br>Protection work<br>Recall training/off-leash training<br>Basic obedience training<br>Other
